# Infectious and Noninfectious Acute Pericarditis in Children: An 11-Year Experience

**DOI:** 10.1155/2018/5450697

**Published:** 2018-11-08

**Authors:** Nahed Abdel-Haq, Zeinab Moussa, Mohamed Hani Farhat, Leela Chandrasekar, Basim I. Asmar

**Affiliations:** ^1^Division of Infectious Diseases, Children's Hospital of Michigan, Detroit, MI, USA; ^2^Carman and Ann Adams Department of Pediatrics, Wayne State University, Detroit, MI, USA

## Abstract

**Objective:**

The study was undertaken to determine the etiology, review management, and outcome in children diagnosed with acute pericarditis during 11 years at tertiary pediatric institution.

**Methods:**

Retrospective chart review of children diagnosed between 2004 and 2014. Patients with postsurgical pericardial effusions were excluded.

**Results:**

Thirty-two children were identified (median age 10yr/11mo). Pericardiocentesis was performed in 24/32 (75%) patients. The most common cause of pericarditis was infection in 11/32 (34%), followed by inflammatory disorders in 9 (28%). Purulent pericarditis occurred in 5 children including 4 due to* Staphylococcus aureus*: 2 were methicillin resistant (MRSA). All patients with purulent pericarditis had concomitant infection including soft tissue, bone, or lung infection; all had pericardial drain placement and 2 required pericardiotomy and mediastinal exploration. Other infections were due to* Histoplasma capsulatum* (2),* Mycoplasma pneumoniae* (2), Influenza A (1), and Enterovirus (1). Pericarditis/pericardial effusion was the initial presentation in 4 children with systemic lupus erythematosus including one who presented with tamponade and in 2 children who were diagnosed with systemic onset juvenile inflammatory arthritis. Tumors were diagnosed in 2 patients. Five children had recurrent pericarditis. Systemic antibiotics were used in 21/32 (66%) and prednisone was used in 11/32 (34%) patients.

**Conclusion:**

Infections remain an important cause of pericarditis in children. Purulent pericarditis is most commonly caused by* Staphylococcus aureus* and is associated with significant morbidity, need of surgical intervention, and prolonged antibiotic therapy. Echocardiography-guided thoracocentesis remains the preferred diagnostic and therapeutic approach. However, pericardiotomy and drainage are needed when appropriate clinical response is not achieved with percutaneous drainage.

## 1. Introduction

The most common identifiable causes of acute pericarditis in children are bacterial infections, viral pericarditis, inflammatory or connective tissue diseases, malignancies, metabolic diseases, and postpericardiotomy syndrome. In a substantial proportion of cases an etiology of pericardial effusion cannot be determined. Such idiopathic effusions are presumed to be viral or postviral in etiology [1–4]. Bacterial pericarditis occurs during the course of bacteremia. Most cases of bacterial pericarditis have been due to* Staphylococcus aureus* and* Haemophilus influenzae *type b (Hib) prior to including Hib vaccine in routine childhood immunization [1, 5]. Other less common bacterial pathogens are* Neisseria meningitides*,* Streptococcus pneumoniae*, group A* Streptococcus*, and* Salmonella* [1, 6–8].

Pericardiocentesis is indicated in symptomatic patients or when the etiology is in doubt. It is also essential in patients with suspected tamponade [1]. Because pericarditis in young children may be caused by bacterial infections, antibiotics are frequently started until the cause is identified.

Most studies of acute pericarditis in children have been limited to case series of purulent pericarditis or pericarditis with large effusions. The proportion of cases caused by different etiologies including infectious and inflammatory is unclear. The introduction of pneumococcal conjugate vaccines (PCV-7 in 2000 and PCV-13 in 2010) caused a reduction of pneumococcal invasive infections including pneumonia [9, 10]. In addition, there has been an emergence of methicillin-resistant* Staphylococcus aureus* (MRSA) infections in many USA communities including ours since 2000 and the proportion of pericarditis cases caused by MRSA in not clear [11]. Thus it is important to reevaluate the epidemiology of acute purulent pericarditis in the era of community acquired- (CA-) MRSA infections and pneumococcal vaccines.

The purpose of this study is to investigate the changes in the etiology of acute pericarditis as well as review the clinical presentation, management and complications of acute pericarditis at our tertiary pediatric medical center during an 11-year period.

## 2. Patients and Methods

The medical records of children with acute pericarditis diagnosed at Children's Hospital of Michigan during the period January 2004 through December 2014 were reviewed. We excluded patients who had cardiac surgery and all those diagnosed with postpericardiotomy syndrome. The diagnosis of acute pericarditis was established on the basis of clinical manifestations and evidence of pericardial effusion by echocardiogram. We reviewed the predisposing factors, clinical features and the etiology of pericarditis. We also evaluated the utility of imaging and reviewed treatment and clinical outcome.

Pericardial fluid from patients who underwent pericardiocentesis was analyzed for cell count, protein, and LDH content as well as cultures. Cultures from blood and other sites of infection including pleural fluid, wounds, abscesses, or bone aspirates were sent as appropriate when an infectious etiology was suspected. A patient was considered to have purulent pericarditis if the pericardial fluid culture grew a bacterial pathogen or purulent material was drained. Children who presented with clinical findings suggestive of a collagen vascular disease had appropriate laboratory studies performed. Pericardial fluid was sent for cytopathology for patients with suspected malignant effusions.

## 3. Results

The total number of patients was 32 including 21 females and 11 males. Age range was 1 day to 17 years with a median of 10 11/12 years. The most common presenting signs were tachycardia in 29/32 (90%), tachypnea in 22/32 (69%), and fever in 12/32 (38%). Muffled heart sounds were noted in 10/32 (32%) at initial presentation and friction rub was noted in 6/32 (19%) of patients. Jugular venous distension was noted in 5/32 (16%) and hepatic enlargement was documented in 6/32 (19%) of patients.

The etiology of pericardial effusion among the 32 study patients included 11 infections, 9 systemic inflammatory diseases, 2 tumors/malignancy, 2 cardiomyopathies, and 6 undetermined etiologies including 2 with underlying malignancy (Table 1). In patients with purulent pericarditis, there was preceding or concurrent soft tissue abscesses in 2 children with methicillin-susceptible* Staphylococcus aureus* (MSSA) pericarditis; osteomyelitis with subperiosteal abscess; and pulmonary septic emboli in a child with MRSA pericarditis and multifocal pneumonia and empyema in another child with MRSA pericarditis. A child with purulent pericarditis and negative cultures who had received antibiotics prior to surgery had concomitant pneumonia and pleural empyema. The clinical features and pericardial fluid findings of patients who had pericarditis due to infectious causes are summarized in Tables 2 and 3.

An inflammatory etiology was determined or suspected in 9 patients: 2 with newly diagnosed systemic-onset juvenile idiopathic arthritis (JIA) and 5 with systemic lupus erythematosus (SLE) including 4 that were newly diagnosed. A 10-year-old girl, with newly diagnosed SLE, presented with cardiac tamponade. Two children who were diagnosed with pericarditis of undetermined inflammatory etiology were treated with steroids including a child with sickle cell disease with elevated antinuclear antibodies and another with recurrent pericarditis.

Other etiologies included tumors with secondary pericardial invasion in two patients: one due to mediastinal B- cell lymphoma and one due to xanthogranuloma of the epicardium who was previously reported [12]. One child with pancytopenia and macrophage activation syndrome (MAS) was suspected of having infectious pericarditis. Two children with underlying malignancy including one with Hodgkin's disease in remission and one with acute lymphoblastic leukemia (ALL) had suspected viral pericarditis.

There were 8 children with pericardial effusions of unclear etiology, 4 of whom underwent pericardiocentesis with their findings summarized in Table 4. Three of the remaining 4 children had presumed viral pericarditis and one with HIV infection had suspected uremic pericarditis.

Chest radiographs and echocardiograms were performed on all patients. Cardiomegaly was a constant feature in all patients. Apart from pericardial effusions, all patients had normal cardiac anatomy except 2 who had atrial septal defect. Electrocardiograms (EKGs) were performed in 22 patients of whom 16 had abnormal tracing including decreased voltages in 6, raised ST segment in 7 and 3 had T-wave inversion. EKG changes were most notable in the inferior and lateral leads. Pericardiocentesis was performed in 24/32 (75%) patients. These were patients who had clinical or echocardiographic features of suspected tamponade, large effusion, suspected purulent pericardial effusion, positive blood culture or clinical bacterial sepsis, need of intensive care, suspected malignant effusion, and pericardial effusions of uncertain etiology. Pericardial fluid analysis results for infectious and noninfectious etiologies are shown in Tables 3 and 4.

Computed tomography (CT) scan of the chest was part of the clinical evaluation in 15/32 patients. Among those, 9 had infections with CT findings of pneumonia/empyema including 4 who required surgical drainage. Two had lung lesions and mediastinal lymph node inflammation due to* Histoplasma* infection. Details of CT findings in patients with infections are shown in Table 2. Among patients with etiologies other than infections, one had a mediastinal mass by CT scan and was subsequently diagnosed with mediastinal B-cell lymphoma and another had mediastinal adenopathy and was diagnosed with systemic onset JIA. Magnetic resonance imaging (MRI) was obtained in a third patient who was diagnosed with epicardial xanthogranuloma.

Five of the 32 patients had recurrent pericarditis: one was diagnosed with hisptoplasmosis, one with* Mycoplasma pneumoniae* infection, one with JIA, one with restrictive cardiomyopathy, and one with undetermined inflammatory etiology.

Systemic antibiotics were given to 21/32 patients including 10/11 patients with pericarditis due to a documented infection. The most common initial antibiotic regimen was intravenous ceftriaxone and vancomycin. Antibiotics were continued following discharge in 15/32 patients including 6 who received intravenous therapy. Definitive antibiotic therapy given to patients with documented infections is shown in Table 2. Prednisone was given to 11/32 patients including 8 patients with definite or suspected inflammatory etiology at time of discharge. Hydroxychloroquine was given concomitantly with steroids in 5 patients with SLE. Nonsteroidal anti-inflammatory therapy was given solely or in combination with steroids or antibiotics in 7 patients. Surgery was performed in 7 including excision of intrapericardial xanthogranuloma, pericardial tissue biopsy in a child with restrictive cardiomyopathy, and 5 patients with purulent pericarditis who underwent drainage procedures including pericardiotomy and mediastinal exploration for mediastinal fluid drainage and removal of adhesions in 2 patients (Table 2). Two patients died: one with MAS and a preterm neonate with cardiomyopathy. Pericarditis and pericardial effusions improved in the remainder of the patients. Two patients with* Staphylococcus aureus* pericarditis developed hypertension with no clear etiology and were prescribed antihypertensive therapy at time of discharge.

## 4. Discussion

In our study the most common cause of acute pericarditis was infection accounting for 11/32 (34%) of cases, followed by inflammatory causes in 9/32, (28%). Infectious etiologies were varied (Table 2): 5 were bacterial infections including 4 due to* Staphylococcus aureus*. Primary purulent pericarditis is rare in children but often is associated with infection at another site resulting in either direct extension of pulmonary infection or hematogenous spread from a distant site to the pericardium [1, 4, 13]. Certain factors may predispose patients for purulent pericarditis such as immunosuppression, malignancy, preexisting pericardial disease, or previous cardiac surgery [14]. However, all patients with purulent pericarditis in our study had no predisposing conditions or immunodeficiency except one with MSSA pericarditis who was known to have SLE and was receiving prednisone at time of presentation (Table 2).

All our patients with* Staphylococcus aureus* pericarditis were bacteremic and the primary infection was soft tissue abscess or osteomyelitis with hematogenous spread to the pericardium. Concomitant lung infection was present in 3/4 patients including one who had pulmonary septic emboli secondary to MRSA osteomyelitis. However, in another patient with MRSA psoas abscess direct spread from the lung to the pericardium may have occurred following development of pneumonia and empyema adjacent to the pericardium. MRSA pericarditis is rarely reported in adults and children. Among our patients with* S. aureus* pericarditis, 2/4 (50%) were infected with CA-MRSA. Both patients had MRSA bacteremia, one with psoas muscle abscess and the other with osteomyelitis. This is consistent with the increase in CA-MRSA invasive disease that has been noted nationally in the last two decades as well as with what we have noted in our community [11, 15]. Lutmer et al. reported two children with CA-MRSA pericarditis who presented with septic shock and multiorgan failure but improved with treatment [16]. All our patients with* S. aureus* including MRSA pericarditis improved. However, two developed hypertension of unclear etiology.

Two of our study patients developed pericarditis due to* Histoplasma capsulatum*. Both had mediastinal histoplasmosis with lymph node enlargement. Histoplasma pericarditis occurs in 6-10% of patients with acute histoplasmosis, predominantly in younger patients [17, 18]. In endemic areas up to 25% of acute pericarditis cases are caused by histoplasmosis [19]. Most such cases are believed to be caused by a local inflammatory or immune response to adjacent mediastinal infection. Patients typically present with acute pericarditis symptoms including chest pain and dyspnea [20]. Concomitant pleural effusion may develop. The pericardial fluid is frequently hemorrhagic but tamponade is uncommon. The disease process is self-limited in the majority of patients. Recurrences may occur; however progression to constrictive pericarditis is unusual [18]. Most patients respond to anti-inflammatory therapy and antifungal treatment is not routinely indicated unless steroid therapy is considered [21]. Both of our patients received itraconazole treatment as one was receiving concomitant steroids and the other had recent history of chemotherapy for acute lymphoblastic leukemia.

Other documented infections that were associated with pericarditis included 2* Mycoplasma pneumoniae* infections, one influenza A 2009 H1N1 infection and one enterovirus infection. Both children with* M. pneumoniae* infection had positive* Mycoplasma* IgM. Although IgM may be falsely positive, the two patients had concomitant radiological evidence of pneumonia consistent with* Mycoplasma* infection. Both improved with oral azithromycin therapy (Table 2). Cardiac involvement such as pericarditis, myocarditis and myopericarditis is a rare complication of* M. pneumoniae* infection. The incidence ranged from 1% to 8.5% in patients with serological evidence of infection and is more common in adults than in children [22–24]. Pericarditis may occur due to direct invasion, hematogenous seeding or spread via bronchiolar lymphatics. However, as other extrapulmonary manifestations, it may be due to autoimmune mechanism as suggested by the frequency of cross reaction between human antigens and* M. pneumoniae* [22]. Bloody or serosanguinous effusions were noted in both of our patients similar to other reports [23].

Although viral infections are the most common cause of pericarditis in children with enteroviruses being the most frequent, influenza H1N1 pericarditis is extremely rare. Myocarditis has been reported during the course of influenza A infection. The clinical manifestations of myocarditis are mild but there have been reports of fatal cases [25]. However, rare cases of pericarditis and cardiac tamponade have been reported in adults and children [26, 27]. Our patient had underlying medical conditions including Down syndrome with asthma and has been receiving thyroid hormone replacement therapy for hypothyroidism. She improved following pericardiocentesis and oseltamivir treatment as well as a short course of prednisone given for asthma exacerbation. Among our patients, only one otherwise healthy child was diagnosed with enterovirus pericarditis. Because testing for enterovirus by PCR was not routinely done on all patients, it is possible that other cases of unknown etiology were due to enterovirus infection. In contrast to pericarditis reports from other countries, none of our patients was diagnosed with tuberculous pericarditis [28, 29].

Collagen vascular diseases may also cause pericarditis in children [2, 30, 31]. Pancarditis is a known complication of acute rheumatic fever [32]. Pericarditis is a common manifestation of juvenile rheumatoid arthritis and the most common cardiac complication of systemic lupus erythematosus [30, 31, 33]. In our study, pericardial effusions were diagnosed at time of presentation of 2 patients with systemic onset JIA and 4 with SLE, including one who had cardiac tamponade; a rare initial presentation of childhood SLE [34]. Surgical drainage is generally not needed in patients with collagen vascular disease and improvement occurs with medical therapy alone [33]. However, pericardiocentesis was performed in 4 of these patients due to large effusions and for diagnostic work up. All of these patients were treated with anti-inflammatory agents.

Pericardial effusions may develop secondary to tumors either due to direct invasion of the pericardium or due to metastasis [35]. Hodgkin's disease, lymphoma, and leukemia are among the most common malignant tumors that are associated with pericardial involvement [36, 37]. In our study, one patient had B-cell lymphoma and another had epicardial juvenile xanthogranuloma that was treated with surgical resection only. Juvenile xanthogranuloma is a rare histiocytic disorder that predominantly affects the skin. Extracutaneous and systemic forms of the disease are rare [38]. Cardiac involvement has been documented in a few case reports [12, 39, 40]. Solitary lesions may be treated with surgical resection, but chemotherapy and/or radiotherapy have been used in patients with systemic disease [41, 42].

Management of children with acute pericarditis is determined by the severity of symptoms and the underlying cause. Drainage of pericardial fluid is frequently needed in patients with large effusions or tamponade or if a neoplastic process is suspected [2, 35]. In addition to antibiotic therapy, pericardial drainage is essential in management of children with purulent effusions [2, 5, 13]. When pericardiocentesis is performed, fluid should be sent for cell count and appropriate cultures. In certain situations, such as bacterial infection, a pericardial window or pericardiotomy may be required [2, 5, 16]. In our study, 5 children with purulent pericarditis underwent surgical drainage including pericardiotomy.

Antibiotic therapy is frequently given pending cultures and was given to 21/32 (66%) of our patients. Antibiotic treatment of purulent pericarditis should be directed against the most likely causative organisms. It is prudent to obtain cultures early in the course of management to direct definitive antibiotic therapy. The optimal empiric antibiotic treatment of acute purulent pericarditis in children is not well defined. The emergence of CA-MRSA makes the antibiotic choice more challenging. Our empiric antimicrobial therapy for suspected purulent pericarditis is vancomycin for coverage of MSSA/MRSA (including clindamycin-resistant strains) as well as ceftriaxone for coverage of Gram-negative organisms while awaiting cultures and susceptibility results. The vast majority of CA-MRSA strains at our institution belong to the USA300 clone and are typically resistant to erythromycin and susceptible to clindamycin and trimethoprim/sulfamethoxazole [11, 15]. Clindamycin was used successfully to treat pericarditis due to CA-MRSA in our patients.

Nonsteroidal anti-inflammatory medications such as ibuprofen and naproxen are frequently used in patients with viral or idiopathic pericarditis to alleviate chest pain and decrease inflammation [43]. Symptoms typically resolve in a few days of treatment.

The use of steroids in patients with idiopathic pericarditis is controversial. Concerns include reactivation of underlying infection and possible increase risk of recurrence. Therefore, its use is reserved for patients with recurrent pericarditis unresponsive to other anti-inflammatory therapy and to those with suspected or definite rheumatologic disease [44, 45]. Colchicine has been used in patients with recurrent idiopathic or inflammatory pericarditis [46].

Complications of acute pericarditis include cardiac tamponade, recurrence, and constrictive pericarditis [34, 44, 47]. Cardiac tamponade occurs when excessive pericardial fluid leads to increase intrapericardial pressure causing impaired cardiac filling and reduced cardiac output. This will require emergency treatment and pericardial fluid drainage [2, 44, 45]. Tamponade was diagnosed in two of our patients at presentation including a 10yr old with SLE and an infant with epicardial xanthogranuloma extending to the pericardial space. Recurrence of pericarditis is likely due to an immune mediated process or inadequate treatment. In our patients 5 had recurrences among whom 2 with infections (*Histoplasma capsulatum *and* Mycoplasma pneumoniae*) and one with subsequent diagnosis of JIA who responded to steroid therapy. Constrictive pericarditis is the end stage of acute or chronic inflammation that leads to thickened adherent pericardium that impairs ventricular filling [44, 45]. None of our surviving patients developed constriction.

Limitations of our study include its retrospective nature and being from a single center.

## 5. Conclusions

Given the decrease in incidence of invasive Hib and pneumococcal infections following the introduction of Hib and pneumococcal vaccines,* S. aureus* should be considered as the main cause of acute purulent pericarditis in children.* S. aureus* pericarditis should be suspected in children who are clinically very ill or septic appearing and most importantly in those with pericardial involvement in the setting of concomitant skin/soft tissue or musculoskeletal infection.

*Histoplasma* pericarditis should be considered in patients with pericardial effusion and CT findings of mediastinal adenopathy especially in children who live in endemic areas.

In contrast, noninfectious pericarditis should be suspected in children with pericardial effusion who are not acutely ill or septic-appearing. An inflammatory etiology such as systemic JIA or SLE should be considered in patients who present with pericardial effusions, have prolonged fever with elevated inflammatory markers, and have negative work-up for infectious causes.

Empirical treatment of a fully immunized child with purulent pericarditis should include an agent effective against* S. aureus* including MRSA especially in communities where this resistant organism is prevalent such as ours. We recommend a combination therapy of vancomycin plus a third-generation cephalosporin. A third-generation cephalosporin such as ceftriaxone can provide adequate empirical coverage for Gram negatives and Streptococci including breakthrough infections with penicillin-resistant* S. pneumoniae.*

Purulent pericarditis is associated with significant morbidity, requirement of surgical intervention, and prolonged antibiotic therapy. Echocardiography-guided pericardiocentesis remains the preferred diagnostic and therapeutic approach. However, pericardiotomy and drainage are needed when appropriate clinical response is not achieved with percutaneous drainage.

## Figures and Tables

**Table 1:** Causes of acute pericarditis in 32 children (2004-2014).

Etiology	Frequency
Infectious (11)	
*Staphylococcus aureus *	4 (2 MSSA; 2 MRSA)
*Histoplasma capsulatum*	2
*Mycoplasma pneumoniae*	2
Enterovirus	1
Influenza A	1
Purulent (negative cultures)	1

Non-Infectious (21)	
Systemic lupus erythematosus	5
Juvenile idiopathic arthritis	2
Undetermined inflammatory cause	2
B-cell non-Hodgkin's lymphoma	1
Epicardial xanthogranuloma	1
Cardiomyopathy	2

Undetermined (2 with underlying malignancy)	6

Total	32

MSSA: methicillin-susceptible *Staphylococcus aureus.* MRSA: methicillin-resistant *Staphylococcus aureus*.

**Table 2:** Clinical characteristics of 11 children with acute pericarditis due to infectious causes.

Age	Presentation	Associated condition	Relevant labs	Imaging	Etiology	Treatment^*∗*^	Outcome
16yr M	Retrosternal chest pain	SLE and hypertension receiving prednisone 30mg BID. Left hand abscess	Blood culture: MSSA. Left hand abscess: MSSA	Chest CT: Pericarditis with effusion. Bilateral pleural thickening	MSSA	Pericardiocentesis with pericardial drain IV oxacillin x 3 weeks plus Oral rifampin x 2 weeks	Improved

16 mo F	Fever, septic shock, right index finger abscess, tachycardia, respiratory distress	Abscess right index finger	Finger abscess, blood culture: MSSA Pericardial fluid and tissue cultures: MSSA	Chest CT: Pericardial and pleura effusions Echocardiogram: day 1: small pericardial effusion, day 3: large effusion, atrial collapse with systole, tamponade, fibrous strands. Pericardial biopsy: fibrinous pericarditis	MSSA	Pericardiocentesis with drain on day 3: 90 ml of serous fluid drained followed 4 days later by pericardial window with mediastinal exploration and chest tube placement due to fibrosis IV oxacillin x 3 weeks IV cefazolin x 3 weeks	Improved but developed severe hypertension of unclear etiology

6 yr F	Fever, dizziness, right leg swelling, tenderness and induration, respiratory distress, signs of septic shock	Salter-Harris fracture type I right tibia. Osteomyelitis with subperiostal abscess of the right distal tibia	Cultures: blood, wound, urine, pericardial: MRSA. Normal immune work up including oxidative burst assay	Chest CT: multiple nodular cavitary lesions suggestive of septic emboli, left lower lobe pneumonia with effusion, large pericardial effusion	CA-MRSA	Pericardiocentesis, left chest tube placement IV vancomycin and clindamycin x 2 weeks IV clindamycin x 4 weeks	Improved but developed hypertension of unclear etiology

2 yr F	Fever, oral ulcers: herpangina, dyspnea, cough, tachycardia, poor oral intake	Eczema. Multifocal pneumonia with empyema. Right retroperitoneal and psoas abscess	Cultures: blood, pleura fluid, psoas abscess: MRSA. Pericardial biopsy: organizing fibrinous pericarditis	Chest CT: Multifocal pneumona, empyema at right medial pleural space adjacent to heart, pericardial effusion	CA-MRSA	Pericardiocentesis. Incision and drainage of pleural empyema. IV clindamycin and IV vancomycin x 19 days then IV clindamycin to complete 6 weeks	Improved

3 yr F	Fever, respiratory distress, decreased activity	Left lower lobe pneumonia, empyema	Blood, pleural and pericardial fluid cultures: negative Received antibiotics prior to cultures.	Chest CT: left lower lobe pneumonia, large left pleural effusion, pericardial effusion	Culture negative purulent pericarditis	Mediastinal exploration. Pericardiotomy and drainage of pericardial and pleural empyema IV ceftriaxone and vancomycin x 6 weeks	Improved

15y M	Fever, chest pain & pressure. Pericardial rub, tender RUQ	None	*Histoplasma* M band positive	Chest CT: left upper lobe infiltrate, left hilar adenopathy, pericardial, left pleural effusion	Histoplasma capsulatum	Itraconazole x 2 months Ibuprofen	Improved

14 yr M	Right shoulder pain, cough	ALL last chemotherapy 8mo earlier	*Histoplasma* M band positive. CF yeast titer 1:32 (NL 1:8)	Chest CT: patchy left upper lobe infiltrate, mediastinal adenopathy, necrotic left mediastinal LN	Histoplasma capsulatum	Itraconazole x 6 months	Improved Follow up CT chest: resolution of adenopathy, small calcified left upper lobe lesion

2 yr F	Fever, dyspnea	None	Pleural fluid bacterial and viral cultures are negative. Mycoplasma IgM positive	Chest X-ray: cardiolmegaly due to pericardial effusion. Obscured left hemidiaphragm due to possible pneumonia	Mycoplsma pneumoniae	Pericardiocentesis: 360 ml of bloody fluid IV ceftriaxone x 4 days Oral azithromycin x 10 days	Improved

9 yr F	2 hospitalizations: Fever, cough, dyspnea, chest pain, abdominal pain, tachycardia, left lung rales	Left lower lobe pneumonia	Pericardial fluid bacterial, viral, mycobacterial cultures negative. Fluid Enterovirus PCR and Adenovirus PCR are negative. Mycoplasma Ig M positive	Chest CT scan (second hospitalization): pericardial effusion, pericardial thickening, small bilateral pleural effusion, left lower lobe pneumonia	Mycoplsma pneumoniae	Pericardiocentesis during first admission First hospitalization: IV ampicillin followed by IV vancomycin total 5 days, discharge to continue with amoxicillin. Second hospitalization: azithromycin and steroids	Readmitted for recurrence 5 days after initial discharge Improved after second hospitalization

7 yr M	Fever, abdominal pain, sore throat, vomiting, diarrhea, skin rash, tachypnea, hepatomegaly	None	Viral culture: rectum, throat, pericardial fluid: Enterovirus. Pleural fluid Enterovirus PCR positive	Chest CT: pericardial effusion, small right pleural effusion, no mediastinal adenopathy or lung lesions	Enterovirus	Pericardiocentesis	Improved Repeat echocardiogram after drainage showed resolution of effusion

6 yr F	Fever, cough, runny nose, dyspnea, wheezing, tachycardia	Down Syndrome, bicuspid aortic valve, asthma, hypothyroidism,	Influenza PCR +ve 2009 H1N1. Bacterial cultures negative	Echocardiogram: right atrial collapse, pre-tamponade	Influenza A	Pericardiocentesis Oseltamivir, perdnisolne	Improved Repeat echocardiogram after drainage showed resolution of effusion

^*∗*^All patients had drainage via pericardiocentesis for moderate or large pleural effusions.

M: male. F: female. CT: computed tomography. ALL: acute lymphoblastic leukemia. MSSA: methicillin susceptible *Staphylococcus aureus*. CA-MRSA: community-acquired methicillin resistant *Staphylococcus aureus*. SLE: systemic lupus erythematosus.

**Table 3:** Pericardial fluid analysis in children with acute pericarditis due to infection.

Etiology	Fluid appearance	RBC/mm^3^	WBC/mm^3^ (Differential)	LDH (mg/dl) (fluid/serum)	Protein (gm/l) (fluid/serum)	Glucose (mg/dl)
MSSA	Turbid/straw	750	13,450 (N92%)	9417/NA	4.8/6.1	19
MSSA	Serous/turbid fluid	8,800	1815 (N92%)	NA	3.6	106
CA-MRSA	Opaque/bloody	94,250	94,200 (N 86%)	1161/320	3.8/6.4	100
CA-MRSA (pleural fluid/fibrinous pericarditis)	Hazy/straw	350	710 (N 81%)	2801/NA	2.9	33
Purulent pericarditis with negative cultures	Bloody	108,500	7850 (N 99%)	NA	NA	NA
Histoplasma	Opaque/bloody	162,250	1540 (N50, L45%)	1820/336	6.1	85
Histoplasma	Bloody	550,000	1933 (N21, L 55%)	944/135	5.8/6.7	62
Mycoplsma pneumoniae	Bloody	2,219,125	6985 (N11, L 82%)	925/NA	5.1	41
Mycoplsma pneumoniae	Cloudy/bloody	27750	9750 (N89, L4%)	1480/NA	4.9	91
Enterovirus	Bloody/turbid	737,160	1050 (N70, L24%)	1228/287	5.0/6.0	56
Influenza A	Clear/yellow	None	54 (N55, L45%)	94/NA	2.8	104

N: Neutrophil. L: lymphocyte. NA: not available. MSSA: methicillin susceptible *Staphylococcus aureus*. CA-MRSA: community-acquired methicillin resistant *Staphylococcus aureus*.

**Table 4:** Pericardial fluid analysis in children with acute pericarditis due to noninfectious causes.

Etiology	Fluid appearance	RBC/mm^3^	WBC/mm^3^ (Differential)	LDH (mg/dl) (fluid/serum)	Protein (gm/l) (fluid/serum)	Glucose (mg/dl)
SLE	Cloudy/bloody	170,000	3125 (N87, L 3%)	1628/NA	7.2/8.5	61
SLE	Turbid/bloody	66,750	1500 (N97%, L 2%)	564/NA	6.1/NA	46
SLE	Clear/yellow	202	143 (N92%)	114/NA	5.2/NA	93
JIA	Cloudy/bloody	40,250	245 (N27, L70%)	231/134	5.8	NA
Undetermined inflammatory ^a^	Cloudy/amber	4,950	4410 (N 57%, L 9%)	454/200	6.4	94
ALL: unknown etiology ^b^	Clear/yellow	106	12 (N17, L 66%)	NA	4.9/5.4	87
Mediastinal large B-cell non-Hodgkin's lymphoma ^c^	Cloudy/yellow	750	4375 (N2%, L 97%)	2618/782	5.5	NA
Epicardial juvenile xanthogranuloma ^d^	Turbid/bloody	241,500	2 (N 46%, L 15%)	269/215	3.7/4.7	72
MAS ^e^	Bloody	4,000,000	4 (L 89%)	930/NA	4.9	NA
Unknown ^f^	Cloudy/bloody	21,732	12 (L 80%)	107	3.7	77
Unknown ^g^	Cloudy/yellow	261	765 (N 63%, L 37%)	204/137	5.6/7.6	93
Unknown ^h^	Cloudy/straw	657	38 (N50, L 13%)	101	< 2/3.9	166
Cardiomyopathy ^i^	Yellow	220	37 (N 49%, L 5%)	184	<2	58

N: Neutrophil. L: lymphocyte. NA: not available. JIA: juvenile idipathic arthritis. SLE: systemic lupus erythematosus. ALL: acute lymphoblastic leukemia.

^a^ 17-yr-old boy with sickle cell disease, antinuclear antibody (ANA) positive.

^b^ 9-yr-old boy; cultures negative. EBV and CMV PCR negative. Had concurrent sinusitis treated with azithromycin: improved.

^c^ 17-yr-old boy with mediastinal mass obstructing the superior vena cava and large pericardial effusion; had pericardiocentesis and given chemotherapy.

^d^ 2-mo-old girl (ex 33 week gestational age); congenital hypothyroidism; diagnosed with large pericardial effusion and tamponade; echocardiogram and MRI revealed a mass; had pericardiocentesis and excision of the mass.

^e^ 20 mo old boy with macrophage activation syndrome. Had heptosplenomegaly, pancytopenia, possible mitochondrial disorder; died

^f^ 15-yr-old girl with massive pericardial effusion with impending tamponade, diagnosed with restrictive cardiomyopathy. Underwent cardiac catheterization and balloon pericardiotomy.

^g^ 14-yr-old girl; cultures negative, enterovirus and Adenovirus PCR negative, ANA negative, and normal complements.

^h^ 6-day-old preterm (24wk gestational age) baby girl with large pericardial effusion: treated for bacterial sepsis with negative cultures, enterovirus PCR negative. Bacterial and mycobactrial cultures negative. ANA negative.

^i^ Newborn preterm (30wk gestational age) baby girl with large pericardial effusion and tamponade; diagnosed with cardiomyopathy; died.

## Data Availability

The data used to support the findings of this study are included within the article.
